# Memory Impairments: Type, Causes, and Molecular Players—Memory Dysfunction Across Neurologic Insults

**DOI:** 10.3390/cells15100923

**Published:** 2026-05-18

**Authors:** Saad A. Farooqui, Maryline Santerre, Natalia Shcherbik, Bassel E. Sawaya

**Affiliations:** 1 FELS Cancer Institute for Personalized Medicine, Lewis Katz School of Medicine, Temple University, Philadelphia, PA 19140, USA; 2 Department of Cell and Molecular Biology, School of Osteopathic Medicine, Rowan University, Stratford, NJ 08084, USA; 3 Department of Cancer and Cellular Biology, Lewis Katz School of Medicine, Temple University, Philadelphia, PA 19140, USA; 4 Department of Neural Sciences, Lewis Katz School of Medicine, Temple University, Philadelphia, PA 19140, USA

**Keywords:** memory impairment, neurodegenerative diseases, viruses, other stimuli

## Abstract

Viral infections of the central nervous system produce memory impairment through mechanisms that extend beyond acute neuronal injury. Herpes simplex virus type 1, human immunodeficiency virus, varicella zoster virus, cytomegalovirus, Epstein–Barr virus, influenza, SARS-CoV-2, West Nile virus, and Zika virus each enter or engage the brain through distinct routes, yet converge on four shared molecular pathways that selectively damage hippocampal circuits: mitochondria-associated membrane (MAM) dysfunction, chronic neuroinflammation, blood–brain barrier (BBB) disruption, and impaired CREB-BDNF signaling. These pathways specifically compromise the dentate gyrus, CA3, and CA1 subfields, producing predictable deficits in pattern separation, associative retrieval, and temporal memory binding. Antiretroviral and antiviral therapies suppress viral replication but fail to reverse organelle-level dysfunction, leaving most hippocampal injury unaddressed. Emerging plasma biomarkers, p-tau217, neurofilament light chain, and GFAP, combined with hippocampal subfield MRI, now enable mechanistic stratification before irreversible circuit loss occurs. This review proposes, as a unifying hypothesis, that virus-associated memory impairment represents a convergent hippocampal syndrome driven by shared downstream pathways, and that combination therapies targeting these pathways simultaneously offer greater therapeutic promise than pathogen-specific approaches alone. The evidentiary basis for this framework varies across pathogens and conditions; direct mechanistic evidence, mechanistic analogy, and preclinical data are distinguished throughout.

## 1. Introduction: Memory Architecture and Its Convergent Vulnerabilities

Memory impairment is among medicine’s most prevalent and least reversible consequences of neurological disease, yet the field has long treated it as a collection of distinct pathogen- or disease-specific syndromes. Alzheimer’s amyloid plaques, Parkinson’s α-synuclein aggregates, HIV viral proteins, stroke-induced ischemia, and post-traumatic stress disorder produce fundamentally different upstream pathologies yet arrive at the same clinical destination: the selective failure of hippocampal circuits that encode, consolidate, and retrieve episodic memory [1,2].

We propose that this convergence is not coincidental but reflects a shared downstream architecture in which diverse triggers engage four common molecular pathways, mitochondria-associated membrane (MAM) dysfunction [3,4], chronic neuroinflammation [5], blood–brain barrier (BBB) disruption [6], and impaired CREB-BDNF signaling [7,8], that selectively compromise hippocampal subfields across at least 26 neurological, viral, psychiatric, and acquired conditions. This framework is presented as a unifying hypothesis intended to motivate combination therapeutic research, and the strength of the supporting evidence is distinguished throughout as direct mechanistic demonstration, mechanistic analogy, or primarily preclinical data.

Understanding why the hippocampus is the convergent target requires a brief account of memory systems and their neural substrates. Memory is not a single faculty but a collection of functionally and anatomically distinct systems, each with different vulnerabilities to disease. Working memory, the temporary maintenance and manipulation of information online, depends principally on the dorsolateral prefrontal and posterior parietal cortices; its capacity is limited to approximately 4 items and degrades under interference or cognitive load [9,10]. Importantly, the hippocampus contributes to working memory under high-load or relational conditions, linking even short-term cognition to the circuit vulnerabilities described in this review [11]. Long-term memory divides into declarative and nondeclarative streams. Declarative memory is explicit and conscious: episodic memory, the retrieval of personally experienced events in their spatiotemporal context, depends on the hippocampal–entorhinal network and is exquisitely sensitive to metabolic, inflammatory, and synaptic disruption [12,13]; semantic memory, the store of factual and conceptual knowledge, is distributed across anterior temporal cortical networks. Nondeclarative memory, procedural skills, habits, priming, and conditioned responses are mediated by corticostriato-cerebellar circuits and remain intact until subcortical and cerebellar systems are also compromised [14,15]. This dissociation is clinically important: procedural memory is typically the last to fail across the conditions reviewed here, while episodic memory is the first and most consistently affected.

At the core of the most demanding memory computations is the hippocampal–entorhinal circuit. The dentate gyrus (DG) performs pattern separation, generating orthogonal representations of overlapping inputs to minimize mnemonic interference [16]. CA3 performs pattern completion through recurrent collateral circuitry, reconstructing memories from partial cues. CA1 integrates hippocampal output with entorhinal temporal signals to encode the sequential structure that gives episodic memory its narrative coherence [17]. Adult neurogenesis in the rodent dentate gyrus supports ongoing pattern separation; its extent in humans remains debated [18], but dentate gyrus plasticity performs analogous functions regardless of its mechanism. These three subfields impose extraordinary metabolic, synaptic, and neurovascular demands: CA1 is the most perfusion-sensitive structure in the brain; the DG neurogenic niche is acutely sensitive to neuroinflammatory suppression; and the hippocampal microvasculature is particularly susceptible to cytokine-mediated BBB disruption. Any pathological process that impairs energy metabolism, vascular integrity, trophic signaling, or immune homeostasis will therefore disproportionately damage hippocampal-dependent episodic memory, precisely what the four convergent pathways do. The subfield-specificity of injury is diagnostically informative: DG damage produces pattern separation failure and encoding deficits; CA3 injury fragments associative recall; CA1 compromise disrupts temporal ordering and context-dependent memory [10,17]. These profiles map directly onto the clinical presentations reviewed in the following sections.

Conditions were included in this review when peer-reviewed evidence supported the involvement of at least two of the four pathways and documented hippocampal subfield involvement. Where evidence is indirect, mechanistically analogous, or primarily preclinical, this is explicitly designated in the text and in the accompanying tables. The goal is not to assert equivalent mechanistic documentation across all 26 conditions, but to provide a scaffold that motivates combination therapeutic strategies commensurate with the complexity of the injury (Table 1).

## 2. Neurodegenerative Diseases and Memory Impairment

Neurodegeneration does not randomly erase memories; it follows predictable anatomical trajectories determined by protein propagation patterns, regional metabolic vulnerabilities, and the selective engagement of the four convergent pathways. Despite heterogeneous molecular triggers, the same hippocampal subfields fail, the same computations degrade, and the same downstream mechanisms converge. What differs across conditions is the entry point; what converges is the outcome.

***A.***  ***Age-Related Neurodegenerative Diseases***

**1. Alzheimer’s Disease (AD).** AD accounts for 60–80% of dementia cases [19]. Amyloid plaques and tau tangles propagate from the entorhinal cortex to the hippocampus according to Braak staging, producing synaptic loss, neuroinflammation, and neuronal death [20]. Early DG and CA1 damage disrupts pattern separation and temporal coding, generating mnemonic discrimination deficits that precede clinical diagnosis [21]; semantic and working memory decline follows, while procedural memory is preserved into late stages [22]. Amnestic MCI progresses to dementia at 10–15% annually [23]; plasma p-tau217/231 and Aβ42/40 predict hippocampal atrophy. All four convergent pathways are engaged: MAM dysfunction via presenilin-mediated ER–mitochondrial decoupling [24]; chronic neuroinflammation driven by Aβ-primed microglia with TREM2-dependent clearance failure [25]; BBB disruption via pericyte loss and MMP-mediated tight junction degradation [26]; and CREB-BDNF suppression by Aβ oligomers. Anti-amyloid antibodies modestly slow progression, reinforcing the need for combination approaches that simultaneously target downstream convergent pathways.

**2. Vascular Dementia (VaD).** VaD accounts for 15–20% of dementia cases and frequently co-occurs with AD [27]. Its subtypes, multi-infarct, small-vessel subcortical ischemic, strategic single-infarct, and hypoperfusion, share the common feature of CA1 vulnerability to ischemic injury [28,29]. Hypoperfusion, oxidative stress, and neuroinflammation converge on encoding and consolidation failure. BBB disruption is the dominant pathway, with white matter hyperintensities reflecting ischemic demyelination and microglial activation, which compound hippocampal injury [28]. MAM pathway involvement is inferred from the shared mitochondrial stress phenotype of ischemic neurons; CREB-BDNF suppression occurs through ischemia-mediated oxidative stress.

**3. Lewy Body Dementia (LBD).** LBD encompasses dementia with Lewy bodies (DLB) and Parkinson’s disease dementia (PDD), distinguished by the one-year rule separating cognitive and motor onset [30]. α-Synuclein aggregates propagate from the brainstem to limbic and cortical regions, often with AD co-pathology [31]. Episodic memory deficits reflect CA1 vulnerability rather than DG failure; retrieval is more impaired than encoding, distinguishing LBD from AD [32,33]. Cognitive fluctuations and visual hallucinations correlate with EEG slowing [34]; REM sleep behavior disorder is a characteristic early sign [35]. DaTscan imaging and the fourth consensus criteria support the diagnosis [30,36]. All four pathways are engaged, with α-synuclein-mediated mitochondrial dysfunction and MAM disruption [3] central to the bioenergetic failure underlying CA1 synaptic loss.

***B.***  ***Idiopathic Neurodegenerative Diseases***

**4. Parkinson’s Disease (PD).** Monogenic forms of PD caused by LRRK2, SNCA, PINK1, PARK2, and GBA mutations share the same convergent pathway engagement described here but represent age-related, genetically defined entry points that are more appropriately classified alongside the genetic neurodegenerative conditions in Sections A and C.

PD affects 1–2% of adults over 65 [37]. Dopaminergic degeneration in the substantia nigra reduces striatal dopamine, while α-synuclein Lewy bodies spread caudorostrally through cholinergic, noradrenergic, and serotonergic systems [38]. Early cognitive deficits reflect frontostriatal dysfunction, impaired working memory and executive control, rather than direct hippocampal damage [39]. Episodic memory shows early retrieval deficits, with recognition preserved; CA1 involvement emerges later; procedural learning fails when internal cues are required [40]. Approximately 80% develop dementia within 20 years, driven by late-stage mitochondrial stress, MAM dysfunction, and CA1 synaptic dysregulation [25].

**5. Progressive Supranuclear Palsy (PSP), Multiple System Atrophy (MSA), Normal Pressure Hydrocephalus (NPH), and Corticobasal Syndrome (CBS).** These conditions produce retrieval-biased memory deficits through disconnection of subcortical and frontostriatal networks rather than direct hippocampal damage. PSP involves tau deposition in the basal ganglia, brainstem, and frontal cortex (MAPT H1 haplotype), impairing working memory and executive retrieval while relatively sparing encoding. MSA produces mild executive and working memory impairment through striato–cerebellar degeneration; hippocampal involvement is secondary. NPH generates gait-dominant frontostriatal compression with reversible cognitive impairment in 60–80% of cases following CSF shunting. CBS presents asymmetric frontoparietal degeneration with visuospatial and working memory failure, frequently with underlying CBD, PSP, AD, or FTLD pathology. Across all four, MAM dysfunction and neuroinflammation are documented (direct or analogous evidence), while BBB disruption and CREB-BDNF impairment are variably supported.

***C.***  ***Genetic Neurodegenerative Diseases***

**6. Huntington’s Disease (HD).** HD results from CAG repeat expansion in HTT, with a prevalence of 5–10 per 100,000 [41]. Medium spiny neuron degeneration in the striatum disrupts cortico–striatal–thalamic circuits before cortical atrophy appears [42]. Mutant huntingtin impairs transcription, axonal transport, and mitochondrial function; reduced BDNF accelerates striatal vulnerability [43,44,45]. Cognitive decline begins years before motor signs, with early working memory deficits reflecting frontostriatal failure and episodic encoding impairment emerging as the hippocampus becomes involved [46]. All four pathways are engaged with strong preclinical evidence.

**7. Spinocerebellar Ataxias (SCAs) and Friedreich’s Ataxia (FRDA).** SCAs are a genetically heterogeneous group (ATXN1/2/3, CACNA1A, and others) producing cerebellar degeneration with secondary cerebellar–frontal circuit disconnection, impairing working memory, procedural learning, and spatial episodic retrieval. FRDA (FXN GAA expansion) leads to mitochondrial iron accumulation and oxidative stress in the cerebellum and spinal cord, with analogous impairments in working memory and procedural learning. MAM dysfunction and neuroinflammation are supported; BBB involvement is limited; CREB-BDNF impairment is documented for FRDA through mitochondrial oxidative stress. Omaveloxolone (an Nrf2 activator) and AAV-frataxin gene therapy represent emerging therapeutic approaches for FRDA.

**8. ALS and Frontotemporal Dementia (FTD).** FTD encompasses behavioral-variant, semantic-variant, and nonfluent primary progressive aphasia subtypes [47], with molecular heterogeneity across FTLD-tau, FTLD-TDP, and FTLD-FUS pathology driven by MAPT, GRN, and C9orf72 mutations [48]. Frontal degeneration impairs strategic retrieval; hippocampal and entorhinal involvement disrupts DG and CA1 functions [49]. Semantic-variant FTD damages semantic memory through anterior temporal atrophy while initially sparing episodic memory [50]. ALS produces working memory and executive impairment through frontal cortical involvement; ALS-FTD overlap (C9orf72) causes combined frontal–hippocampal failure. TDP-43 mislocalization and C9orf72 poly-PR aggregation drive RNA processing failure and MAM disruption [51]. All four pathways are engaged in FTD; direct BBB evidence is more limited in ALS without FTD co-pathology.

**9. Creutzfeldt–Jakob Disease (CJD).** Although prion diseases are classified here under genetic conditions based on PRNP mutation forms, sporadic CJD, the most common presentation, shares mechanistic features with idiopathic neurodegeneration, and the placement reflects molecular rather than etiological classification. CJD is a rapidly progressive prion disease that produces global network failure through widespread propagation of prions in the cortex, thalamus, and cerebellum. All memory systems fail simultaneously within months. The prion protein misfolding cascade impairs MAM function and triggers neuroinflammation; BBB disruption and CREB-BDNF impairment occur as secondary consequences of rapid neurodegeneration. No disease-modifying therapy exists; management is palliative.

***D.***  ***Other Neurodegenerative Conditions***

**10. Korsakoff Syndrome (KS).** KS results from thiamine deficiency, most commonly in the context of chronic alcohol use disorder, producing selective destruction of the mammillary bodies and mediodorsal thalamus through the Papez circuit, with severe anterograde and retrograde amnesia in the context of preserved working memory and procedural learning. Hippocampal–diencephalic disconnection disrupts consolidation without directly damaging the hippocampus. Thiamine-dependent metabolic failure engages MAM dysfunction and neuroinflammation; BBB disruption and CREB-BDNF suppression are documented in the context of chronic alcohol exposure. Thiamine replacement prevents progression if initiated early; established KS shows limited reversal.

**11. Chronic Traumatic Encephalopathy (CTE).** CTE results from repetitive head trauma, producing perivascular tau deposition in frontal and temporal cortices that spreads to the hippocampus with advancing stage. Episodic and working memory impairment, executive dysfunction, and behavioral change emerge years to decades after exposure. All four convergent pathways are engaged: BBB disruption from repeated mechanical injury initiates the cascade; tau propagation disrupts MAM contacts; chronic neuroinflammation sustains microglial activation; and CREB-BDNF impairment compounds hippocampal synaptic fragility. APOE4 accelerates disease course and increases CTE severity following equivalent trauma exposure.

**Conclusion.** Across these 17 neurodegenerative conditions, memory impairment arises at the intersection of hippocampal subfield vulnerability and convergent pathways. Hippocampal-predominant conditions (AD, KS, HSV encephalitis) show early DG/CA1 encoding failure. Subcortical and frontostriatal conditions (PD, HD, PSP, MSA, NPH, VaD) produce retrieval-biased deficits through network disconnection. Frontotemporal conditions (FTD, PPA, ALS, CBS) vary with the balance of frontal versus hippocampal involvement. Cerebellar conditions (SCAs, FRDA) impair working memory and procedural learning. CJD and severe CTE overwhelm all systems simultaneously. Despite this heterogeneity, MAM dysfunction, CREB-BDNF impairment, BBB breakdown, and chronic neuroinflammation recur as the shared drivers, supporting a unified mechanistic framework (Table 2 and Table 3).

## 3. Viral Pathogens and Their Convergent Hippocampal Mechanisms

The nine pathogens discussed below differ substantially in their routes of CNS engagement, the directness of their neuroinvasive effects, and the strength of evidence linking them to the four convergent pathways. Direct neuroinvasive effects, post-infectious inflammatory syndromes, vascular mechanisms, and epidemiological associations are distinguished where evidence permits. Pathway involvement is designated as supported by direct mechanistic evidence, mechanistic analogy to related models, or primarily preclinical data.

1. Herpes Simplex Virus Type 1 (HSV-1)

HSV-1 establishes latency in the trigeminal ganglia following primary infection, with seroprevalence reaching approximately 70% in adults aged 50 years or older [52]. Reactivation allows spread to the hippocampus and entorhinal cortex via olfactory and trigeminal projections, and acute encephalitis produces severe anterograde and retrograde amnesia through hippocampal necrosis. The more clinically prevalent scenario is recurrent subclinical reactivation, which silently impairs encoding and consolidation through sustained synaptic loss and low-grade inflammation [53]. At the molecular level, HSV-1 engages all four convergent pathways. Viral glycoproteins seed amyloid-beta aggregation, HSV DNA is detectable within senile plaques, and reactivation increases beta- and gamma-secretase activity, linking HSV-1 to the same MAM-disrupting presenilin pathology seen in Alzheimer’s disease [54]. Microglial activation during reactivation produces sustained IL-6 and TNF-alpha release that suppresses hippocampal neurogenesis and LTP. HSV-1 glycoprotein B directly disrupts tight junction proteins, compromising BBB integrity. CREB phosphorylation is suppressed during active viral replication through multiple kinase-mediated mechanisms, reducing BDNF expression and synaptic resilience [54,55]. The APOE4 genotype substantially amplifies hippocampal vulnerability to HSV-1, with carriers exhibiting impaired viral clearance, more extensive hippocampal spread, and accelerated amyloid seeding, thereby increasing the risk of Alzheimer’s disease by 1.5–2.5-fold compared with seronegative individuals [55]. The evidence linking HSV-1 to MAM dysfunction and CREB-BDNF suppression is predominantly derived from in vitro and animal models; direct human mechanistic data remain limited, and the causal relationship between subclinical reactivation and progressive hippocampal injury in immunocompetent individuals requires further longitudinal validation.

2. Human Immunodeficiency Virus (HIV)

HIV enters the central nervous system within weeks of systemic infection, establishing reservoirs in perivascular macrophages and microglia that persist despite effective antiretroviral therapy [56]. The consequence is a chronic neuroinflammatory state that drives HIV-associated neurocognitive disorder (HAND) in 30–50% of treated individuals, a prevalence that has not substantially declined in the antiretroviral era, indicating that viral suppression is insufficient to resolve the underlying molecular pathology [57]. The viral protein Tat is released by infected cells and taken up by neighboring neurons regardless of the neurons’ infection status, and it remains detectable in the CNS even under effective antiretroviral suppression [16,18,19,58,59]. Tat activates c-Src kinase, which phosphorylates PTPIP51 at Y176, displacing VAPB from MAM contact sites without altering total protein expression, collapsing ER-mitochondria calcium transfer and driving a lipidomic signature, triglyceride accumulation, membrane phospholipid depletion, ceramide elevation, and bioenergetic failure that mirrors aged brain tissue [3,4]. The resulting metabolic state independently increases ischemic vulnerability, providing a molecular bridge between HIV infection and the 1.5–2-fold elevated stroke risk observed epidemiologically in people living with HIV [10,11]. Estimates of the degree to which HIV accelerates brain aging vary across studies and methodological approaches; the figures cited here reflect observed ranges from epidemiological cohort studies rather than a settled consensus [56,57].

Beyond MAM dysfunction, Tat inhibits CREB phosphorylation and reduces BDNF levels, while gp120 activates caspase-3, thereby compounding CREB-dependent transcriptional failure [60,61]. Both proteins independently compromise BBB integrity by disrupting tight junction proteins and activating matrix metalloproteinases. Microglial activation driven by Tat and gp120 produces sustained IL-6, TNF-alpha, and complement-mediated synaptic pruning that persists under viral suppression [62,63]. The hippocampal consequences are subfield-specific: suppressed dentate gyrus neurogenesis impairs pattern separation, CA1 synaptic loss disrupts temporal encoding, and disrupted DG-CA3 connectivity fragments associative retrieval [62].

3. Varicella Zoster Virus (VZV)

VZV reactivation from sensory ganglia triggers cerebral vasculopathy as a primary mechanism of hippocampal injury, distinguishing it from the direct neurotropic mechanisms of HSV-1 and HIV [64,65]. Infection of cerebral arterial walls produces ischemic lesions, white matter hyperintensities, and antiphospholipid-mediated thrombosis, with CA1 bearing the disproportionate burden of injury. VZV-associated vasculopathy activates the complement cascade and drives endothelial inflammation, compromising tight junction integrity throughout the cerebrovasculature and thereby allowing sustained access of peripheral inflammatory mediators to the hippocampal microenvironment. CREB-BDNF signaling is suppressed through ischemia-mediated oxidative stress and cytokine-driven pathways. The cumulative effect is a 20–30% increased risk of dementia, particularly vascular and mixed subtypes, in individuals with a history of VZV reactivation [64,65]. Direct evidence for MAM dysfunction in VZV-associated hippocampal injury is currently limited; this pathway is included by mechanistic analogy with ischemia-driven MAM disruption documented in other cerebrovascular contexts.

4. Cytomegalovirus (CMV)

CMV achieves near-universal latency in immunocompetent hosts, with seroprevalence reaching 70–90% in elderly populations [66,67]. Its hippocampal impact is mediated primarily through the chronic immune activation state that periodic reactivation sustains over decades. This inflammaging phenotype, characterized by reduced T-cell diversity, chronically elevated IL-6 and TNF-alpha, and impaired immune resolution, erodes hippocampal resilience through sustained microglial activation and progressive BDNF suppression [68,69]. Reactivation-driven cytokines directly impair CREB phosphorylation and reduce BDNF expression, and the BDNF Val66Met polymorphism substantially amplifies this vulnerability by impairing activity-dependent BDNF secretion [68]. The MAM pathway is engaged through sustained mitochondrial stress driven by chronic cytokine exposure and impaired mitophagy. The evidence for direct CMV-induced MAM disruption is predominantly preclinical; the link to hippocampal MAM dysfunction in human subjects has not been directly demonstrated and represents a testable hypothesis generated by the convergent framework.

5. Epstein–Barr Virus (EBV)

EBV establishes lifelong latency in B lymphocytes, and its relationship to hippocampal memory impairment operates primarily through autoimmune and demyelinating mechanisms rather than direct neuroinvasion [70]. EBV nuclear antigen 1 (EBNA-1) contains molecular mimicry sequences that trigger cross-reactive antibody production, damaging hippocampal and frontotemporal networks [71]. In the context of multiple sclerosis, where EBV seropositivity is nearly universal, this autoimmune hippocampal attack contributes to the episodic memory deficits, working memory impairment, and processing speed reductions that characterize MS-associated cognitive decline [72]. The CREB-BDNF pathway is suppressed through EBV-driven cytokine production and through the direct effects of demyelination on hippocampal afferent connectivity. BBB disruption is mediated by EBV-associated lymphocyte infiltration and complement activation. Direct evidence for MAM dysfunction in EBV-associated hippocampal injury is lacking in the current literature; this pathway is considered a mechanistic analogy based on shared downstream mitochondrial stress signatures observed in demyelinating and autoimmune neurological conditions.

6. Influenza Virus

Influenza virus rarely achieves direct CNS entry in immunocompetent individuals, yet severe influenza infection produces cognitive impairment persisting for months to years in 15–30% of cases, predominantly affecting episodic memory, attention, and executive function [73]. The mechanism is a systemic cytokine storm: IL-6, TNF-alpha, and IL-1beta, produced peripherally during the acute infection, breach the blood–brain barrier, producing a neuroinflammatory state that outlasts the period of active viral replication. This post-infectious neuroinflammation inhibits hippocampal neurogenesis, suppresses LTP, and activates complement-mediated synaptic pruning in the dentate gyrus and CA1. Microglia primed by the initial cytokine storm exhibit exaggerated responses to subsequent inflammatory stimuli, a phenomenon that may contribute to accelerated cognitive aging in individuals with a history of severe influenza. BDNF suppression during the acute phase reduces the capacity for hippocampal circuit recovery, while BBB disruption allows ongoing peripheral immune surveillance of the CNS parenchyma. MAM pathway involvement in influenza-associated hippocampal injury is inferred from the shared mitochondrial stress phenotype observed with cytokine-driven neuroinflammation; direct mechanistic evidence in influenza-specific models is limited.

7. SARS-CoV-2

Long COVID cognitive impairment, characterized by memory deficits, processing speed reductions, and attentional dysfunction, affects 10–30% of individuals following SARS-CoV-2 infection [74]. More recent longitudinal cohort studies have refined these estimates and suggest that a meaningful proportion of affected individuals show partial recovery over 12–24 months, though a subset experiences persistent deficit; the literature through 2024 is incorporated here [74,75]. SARS-CoV-2 gains CNS access through the olfactory epithelium and through systemic endothelial infection that compromises BBB integrity throughout the cerebral vasculature. MRI studies demonstrate hippocampal and parahippocampal gray matter loss in individuals with long COVID, and postmortem analysis reveals microgliosis and synaptic loss without widespread viral RNA in brain parenchyma, indicating that the neurological injury is driven primarily by inflammatory rather than direct cytopathic mechanisms [75]. At the molecular level, ongoing microglial activation and complement dysregulation, with elevated C1q and C3 detectable months after viral clearance, drive sustained synapse elimination in the dentate gyrus and CA1 [74,75]. ACE2-mediated endothelial infection compromises tight junction proteins across the cerebral microvasculature. Mitochondrial dysfunction driven by viral N protein-mediated disruption of mitochondrial membrane dynamics impairs MAM calcium transfer and ATP production. CREB-BDNF signaling is suppressed through cytokine-mediated pathways and through direct impairment of ACE2-dependent signaling [76].

8. West Nile Virus (WNV)

West Nile virus neuroinvasive disease directly infects hippocampal neurons, producing acute neuronal loss, astrogliosis, and microglial activation [77,78]. Episodic memory deficits and executive dysfunction persist for months to years following neuroinvasive WNV infection, with ongoing neuroinflammation impairing neurogenesis and synaptic plasticity in the absence of detectable viral replication [79]. The sustained inflammatory response maintains a microglial activation state that progressively erodes CA1 synaptic density and dentate gyrus progenitor cell populations. MAM dysfunction in WNV-infected neurons arises through direct viral disruption of mitochondrial membrane dynamics, with WNV NS5 protein impairing mitochondrial calcium uptake and driving bioenergetic failure that compounds the structural neuronal injury of acute infection. CREB-BDNF signaling is suppressed through NF-kB-mediated inhibition of CREB phosphorylation during acute infection, with persistent BDNF reduction contributing to impaired hippocampal circuit recovery.

9. Zika Virus (ZIKV)

ZIKV exerts its most severe hippocampal effects during adult infection by targeting neural progenitor cells in the dentate gyrus, thereby disrupting the neurogenic niche that supports ongoing pattern separation and hippocampal circuit adaptation [80]. ZIKV directly infects Sox2-positive neural progenitors, inducing cell-cycle arrest and apoptosis, thereby depleting the dentate gyrus progenitor pool. Spatial memory deficits and sustained microglial activation persist for weeks after viral clearance in adult animal models [80]. Microglial activation drives progenitor cell loss through cytokine-mediated suppression of Wnt signaling, BDNF production is reduced through both direct viral effects and inflammatory suppression of CREB-dependent transcription, and BBB disruption in the hippocampal microvasculature amplifies the neuroinflammatory environment during the acute phase. Direct evidence for MAM dysfunction in ZIKV infection is currently absent from the human literature; its inclusion here reflects a mechanistic analogy with progenitor cell metabolic stress phenotypes documented in related flavivirus models (Table 4).

## 4. The Four Convergent Molecular Pathways: Mechanistic Detail

**A1.**  **Mitochondria-Associated Membrane (MAM) Dysfunction**

Mitochondria-associated membranes are specialized contact sites between the endoplasmic reticulum and mitochondrial outer membrane, maintained by protein tethering complexes including VAPB-PTPIP51, that regulate calcium transfer from ER stores to mitochondria, ATP synthesis, lipid metabolism, and autophagosome formation [3,4]. The spatial proximity maintained by these tethers, typically 10–25 nm, is functionally essential: calcium transfer across this gap drives oxidative phosphorylation and sustains synaptic ATP production. When MAM contacts are disrupted, calcium transfer fails, mitochondrial ATP synthesis collapses, and fatty acids are diverted from oxidation and membrane synthesis into triglyceride storage, producing a lipid-loaded, metabolically rigid neuronal state that is acutely vulnerable to additional insults [4]. The hippocampus is exceptionally sensitive to MAM disruption because of the high ATP demands of sustained high-frequency synaptic transmission and the dependence of LTP on calcium-triggered kinase cascades that require MAM-competent mitochondrial buffering. Multiple viral proteins engage this pathway. SARS-CoV-2 N-protein disrupts mitochondrial membrane dynamics and impairs MAM calcium transfer [24,76]. The downstream lipidomic and metabolomic consequences of MAM disruption are consistent across viral and neurodegenerative contexts: triglyceride accumulation with sequestration of arachidonic acid and DHA-containing species, membrane phospholipid depletion, ceramide elevation, and concurrent falls in ATP and acetyl-CoA reflecting mitochondrial bioenergetic failure [4]. This signature mirrors aged brain tissue, providing a molecular basis for the premature cognitive aging observed in HIV and other chronic viral infections. Therapeutic targets at this pathway include sigma-1 receptor agonists, which stabilize MAM contact site integrity, and calcium modulators that restore mitochondrial calcium uptake independently of the tethering complex.

**A2.**  **Chronic Neuroinflammation**

Microglia serve as the brain’s resident immune sentinels, rapidly sensing and responding to pathogen-associated molecular patterns and damage signals. In the context of acute viral infection, this response is protective. However, chronic microglial activation, sustained by persistent viral antigens, ongoing stimulation of pattern recognition receptors by residual viral RNA, and feedback amplification through cytokine networks, transforms the neuroprotective response into a driver of synaptic destruction [5]. Activated microglia eliminate synapses through multiple converging mechanisms: CR3-dependent complement-mediated phagocytosis of C1q- and C3-tagged synaptic terminals, TNF-alpha-mediated reduction in synaptic AMPA receptor expression, IL-1beta-driven suppression of LTP, and direct physical engulfment of dendritic spines. In the hippocampus, this chronic pruning produces progressive circuit degradation that is not reversed by viral clearance or antiretroviral suppression. The TREM2-dependent microglial clearance pathway is relevant across multiple viral contexts: TREM2 loss-of-function impairs microglial debris clearance and amplifies the neuroinflammatory response in Alzheimer’s disease [25], and similar impairments of TREM2-dependent resolution occur in SARS-CoV-2 and HIV-associated neuroinflammation. Complement dysregulation, particularly sustained elevations in C1q and C3, provides a mechanistic link between acute viral neuroinflammation and the progressive synapse loss characteristic of neurodegenerative conditions [74,75]. Therapeutic targets in this pathway include TREM2 agonists that restore microglial homeostasis, NLRP3 inflammasome inhibitors that interrupt IL-1β- driven amplification, and mast cell stabilizers that reduce histamine-mediated BBB permeabilization.

**A3.**  **Blood–Brain Barrier Disruption**

The blood–brain barrier is maintained by the coordinated function of cerebrovascular endothelial tight junctions, pericytes that regulate vascular tone and endothelial integrity, and astrocytic endfeet that couple neuronal metabolic demand to vascular supply [26]. Its disruption exposes CA1, the most perfusion-sensitive and vascularly dependent hippocampal subfield, to systemic inflammation, hypoperfusion, and peripheral immune activation. Viral disruption of BBB integrity occurs through multiple mechanisms: direct endothelial infection (HIV, SARS-CoV-2), cytokine-mediated downregulation of tight junction proteins including claudin-5 and occludin (influenza, CMV, HIV), pericyte loss driven by viral-induced oxidative stress, and complement-mediated endothelial activation (SARS-CoV-2, VZV). Matrix metalloproteinase activity is central to viral BBB disruption: HIV Tat and gp120, SARS-CoV-2 spike protein, and VZV glycoproteins each upregulate MMP-2 and MMP-9, which degrade extracellular matrix components that support endothelial junction integrity and pericyte adhesion. The resulting BBB disruption creates a permissive environment for peripheral monocyte and lymphocyte infiltration, amplifying the neuroinflammatory response. Therapeutic approaches targeting BBB restoration include MMP inhibitors that preserve junctional integrity, pericyte replacement and stabilization strategies, and activation of the angiopoietin-1 pathway, which promotes endothelial junction formation.

**A4.**  **Impaired CREB-BDNF Signaling**

The CREB-BDNF signaling axis is the molecular foundation of hippocampal plasticity and resilience. CREB phosphorylation at Ser133, driven by calcium influx, cAMP elevation, and neurotrophin signaling, activates immediate early gene expression and drives BDNF transcription, which in turn supports dendritic arborization, synaptogenesis, and the maintenance of hippocampal progenitor cell populations [6,7,8]. Suppression of this pathway reduces neurogenesis, impairs LTP, and accelerates hippocampal atrophy. Viral suppression of CREB-BDNF signaling occurs through multiple converging mechanisms. HIV Tat directly inhibits CREB phosphorylation by activating GSK-3beta and interfering with the calcium/calmodulin-dependent kinase cascade [60]. gp120 activates caspase-3, thereby degrading CREB, compounding transcriptional failure [61]. CMV-driven IL-6 and TNF-alpha suppress BDNF through NF-kB-mediated inhibition of CREB-dependent transcription. SARS-CoV-2 impairs ACE2-dependent pathways that normally support trophic signaling in hippocampal neurons. WNV NS5 protein activates NF-kB in a manner that directly competes with CREB for transcriptional co-activator binding [79]. The BDNF Val66Met polymorphism substantially modulates the impact of viral CREB-BDNF suppression, the Met allele impairs activity-dependent BDNF secretion through reduced sorting to the regulated secretory pathway, and carriers show greater hippocampal volume loss in response to CMV reactivation and greater cognitive impairment in HIV [68,81].

An important additional regulatory component within this pathway is TGF-β1, a neurotrophic and cytokine factor whose hippocampal deficit has been consistently associated with depression, cognitive dysfunction, and stress vulnerability in both animal models and clinical studies [82,83]. TGF-β1 SMAD-dependent signaling interacts directly with CREB and modulates dentate gyrus neurogenesis; its suppression during chronic viral neuroinflammation may compound CREB-BDNF impairment, particularly in the context of stress-related comorbidities that frequently co-occur with chronic viral infection. The viral conditions discussed in this review produce neuroinflammatory states that suppress TGF-β1 signaling, and the integration of this pathway component strengthens the mechanistic basis for dentate gyrus neurogenic failure across multiple viral contexts.

*Synergistic Amplification*. These four pathways do not operate in isolation. MAM dysfunction sensitizes hippocampal neurons to inflammatory insults by impairing the bioenergetic capacity required to mount protective responses. BBB disruption floods the hippocampal parenchyma with peripheral inflammatory mediators, amplifying microglial activation. Neuroinflammation elevates ATP demand through cytokine-driven hyperexcitability while simultaneously suppressing BDNF production. CREB-BDNF impairment reduces the expression of PGC-1alpha, the master regulator of mitochondrial biogenesis, completing the cycle by further impairing the mitochondrial function on which MAM integrity depends. This synergistic amplification explains why single-pathway interventions have produced modest cognitive benefit across virtually every memory disorder studied to date, and why combination strategies targeting multiple pathways simultaneously are mechanistically indicated (Table 5).

**B.**  **Genetic Modulators of Viral Hippocampal Vulnerability**

Genetic background substantially modulates the impact of viral engagement of convergent pathways, creating interindividual variation in vulnerability relevant to both risk stratification and the design of precision interventions. APOE4 is the most broadly relevant genetic modifier, amplifying viral hippocampal injury through at least three mechanisms: impaired viral clearance (HSV-1), reduced lipid-mediated neuroprotection in the context of MAM-driven lipid dysregulation (HIV), and potentiated neuroinflammatory responses to complement activation (SARS-CoV-2) [55,76]. APOE4 carriers show accelerated hippocampal atrophy in response to viral infection across multiple pathogen contexts. The BDNF Val66Met polymorphism reduces activity-dependent BDNF secretion and amplifies the impact of viral CREB-BDNF suppression, predicting greater hippocampal volume loss in CMV seropositivity and greater cognitive vulnerability in HIV [68,81]. FKBP5 polymorphisms modulate glucocorticoid receptor sensitivity, influencing the degree to which stress hormones released during acute viral infection amplify hippocampal inflammatory responses. TREM2 variants impair microglial clearance function, prolonging the neuroinflammatory consequences of viral priming.

Sex-dependent differences in hippocampal vulnerability to viral infection represent an understudied but mechanistically important dimension of this framework. Estradiol-mediated protection of MAM tethering reserve, through upregulation of VAPB expression and maintenance of ER-mitochondria contact site density, and estradiol-dependent BDNF expression provide a basis for differential hippocampal vulnerability between sexes observed in HIV-associated neurocognitive disorder and stress-related conditions. These protective mechanisms are attenuated after menopause, consistent with the accelerated cognitive aging and increased HAND severity observed in women over 50. The developmental timing of pathway activation is also relevant: prenatal and perinatal viral exposures engage MAM and neuroinflammatory pathways during critical windows of hippocampal circuit formation, with consequences for neurogenic niche establishment that differ fundamentally from those of adult exposure. Adolescent infection engages pathways during a period of ongoing synaptic pruning and BDNF-dependent circuit refinement, while adult exposure acts on a fully formed but potentially compromised circuit. These developmental distinctions are relevant to interpreting the clinical heterogeneity of post-viral cognitive outcomes across age groups.

**C.**  **Diagnostic Approaches for Virus-Associated Hippocampal Injury**

The convergence of viral hippocampal injury across four shared molecular pathways creates an opportunity for pathway-specific, rather than pathogen-specific, biomarker-guided mechanistic stratification, identifying which component of the convergent injury is driving ongoing dysfunction in each individual. Plasma neurofilament light chain (NfL) provides a sensitive measure of ongoing neuronal injury and axonal damage, rising in proportion to the rate of hippocampal circuit degeneration regardless of the upstream cause [7]. Plasma GFAP reflects astrocytic activation and is particularly sensitive to BBB-associated astrocytic remodeling. Plasma p-tau217 detects tauopathic changes that may be secondary to chronic MAM dysfunction and neuroinflammation, and its elevation in long COVID and HIV-associated neurocognitive decline suggests that tau pathology is a downstream consequence of convergent pathway activation [8]. The CSF/serum albumin quotient provides a direct measure of BBB integrity. Hippocampal subfield MRI, specifically volumetric measurement of dentate gyrus, CA3, and CA1, enables anatomical localization of injury that maps directly onto the functional deficits described above [17].

The degree of validation for these biomarkers is not equivalent across the conditions discussed in this review. NfL and GFAP have the broadest validation across neuroinflammatory and neurodegenerative contexts; p-tau217 as a marker of post-viral hippocampal injury remains an emerging area with limited cross-disorder validation. Hippocampal subfield MRI is predominantly a research tool and has not been validated as a clinical endpoint in post-viral cognitive impairment trials. These tools are presented here as offering a pathway toward precision mechanistic stratification as their cross-disorder validation is established, rather than as currently applicable clinical instruments.

## 5. Psychiatric Disorders and Memory Impairment

Psychiatry and neurology have long been treated as separate disciplines, yet their primary disorders share hippocampal circuits, the same four molecular pathways, and overlapping episodic memory failures. The boundary between a depressed brain and a degenerating one is less categorical than clinical tradition implies.

**1. Major Depressive Disorder (MDD).** MDD affects over 300 million people worldwide; cognitive symptoms, including memory impairment, occur in 85–94% of patients and persist into remission [51]. The memory profile is distinctive: impaired autobiographical encoding, overgeneralized recollection, and reduced pattern separation consistent with DG dysfunction. Meta-analyses confirm an 8–10% reduction in hippocampal volume, with preferential atrophy of CA1 and DG [86]. Chronic glucocorticoid exposure drives mitochondrial dysfunction and impairs ATP production; sustained microglial activation prunes synapses and disrupts BBB integrity; suppressed CREB-BDNF signaling reduces neurogenesis by 30–50% [81]. The BDNF Val66Met polymorphism amplifies risk and predicts greater hippocampal volume loss. SSRIs restore neurogenesis and raise BDNF 2–3-fold; ketamine acts rapidly through BDNF-TrkB signaling and hippocampal synaptogenesis.

**2. Schizophrenia.** Cognitive deficits precede psychosis in 75–85% of individuals and predict long-term functional outcome more reliably than positive symptoms [87]. Working memory impairment is severe (effect size d = 1.0–1.5), with hippocampal volume reduced 4–8% in CA1 and subiculum. Mitochondrial complex I deficits disrupt MAM contacts and energy metabolism; C4A overexpression drives excessive adolescent synaptic pruning through complement-mediated microglial activation [88]; NMDA receptor hypofunction suppresses CREB-BDNF signaling [89]; and BBB disruption elevates the albumin quotient. BDNF Val66Met carriers show greater cognitive impairment. Minocycline improves working memory in early schizophrenia; N-acetylcysteine and aerobic exercise support hippocampal recovery.

**3. Post-Traumatic Stress Disorder (PTSD).** PTSD presents a paradox: traumatic memories are hyperconsolidated and intrusive while context-dependent memory, fear extinction, and pattern separation fail, reflecting DG dysfunction at its core [90]. Hippocampal volume is reduced 5–8%, most prominently in CA3 and DG [91]. Glucocorticoid dysregulation disrupts mitochondrial function and MAM calcium homeostasis; sustained neuroinflammation increases BBB permeability; impaired CREB-BDNF signaling compounds synaptic fragility. BDNF Val66Met increases PTSD risk (OR 1.4–1.8); FKBP5 polymorphisms modulate glucocorticoid receptor sensitivity, predicting hippocampal volume loss [92]. Prolonged exposure therapy increases hippocampal volume; SSRIs enhance BDNF and support fear memory reconsolidation.

**4. Bipolar Disorder.** Cognitive impairment affects 40–60% of individuals even during euthymia [93], with episodic memory deficits (effect size 0.7–1.0) and CA1/DG abnormalities persisting between mood episodes. Mitochondrial complex I deficits correlate with cognitive severity; episodic neuroinflammation during mood episodes elevates IL-6 and TNF-α; BDNF falls during depressive phases, disrupting CREB-dependent plasticity [94]. Lithium is mechanistically coherent: it increases BDNF, enhances mitochondrial function, inhibits GSK-3β, and preserves hippocampal volume [95].

**Convergent Mechanisms.** MDD, schizophrenia, PTSD, and bipolar disorder share a biological rather than symptomatic unity. Genetic vulnerabilities, BDNF Val66Met, FKBP5, COMT, and C4A, translate psychological stress into hippocampal damage through the same four pathways that drive neurodegeneration and viral injury. Combination approaches targeting mitochondrial function, neuroinflammation, BBB integrity, and CREB-BDNF signaling, augmented by aerobic exercise, cognitive remediation, and stress reduction, offer greater promise than neurotransmitter-focused monotherapies. Biomarker-guided stratification using plasma BDNF, inflammatory markers, and hippocampal subfield MRI can enable precision intervention before irreversible circuit loss occurs.

## 6. Additional Factors Affecting Memory

Brain injury, vascular disruption, developmental insults, and neoplastic disease round out the spectrum of memory-impairing conditions. Despite their heterogeneity, they converge on the same hippocampal vulnerabilities through the four shared pathways.

**Traumatic Brain Injury (TBI).** TBI causes diffuse axonal injury, acute BBB disruption, and chronic neuroinflammation that initiate tau pathology and progressive hippocampal circuit failure [96,97]. APOE4 amplifies severity and accelerates post-injury neurodegeneration. Critically, TBI is not an acute event alone: data from the Framingham Heart Study confirm that TBI is associated with increased long-term all-cause mortality driven substantially by dementia-related death [98], underscoring the clinical urgency of targeting convergent hippocampal pathways in TBI survivors. All four pathways are robustly engaged.

**Stroke and Chronic Hypoxia.** Stroke produces focal hippocampal, diencephalic, and frontal lesions compounded by white matter ischemia and suppressed neurogenesis [99]. CA1 is exquisitely sensitive to ischemic injury; even subclinical hypoperfusion impairs encoding and consolidation through mechanisms shared with VaD and metabolic injury. Chronic intermittent hypoxia engages ferroptosis and ER stress pathways in hippocampal neurons [99], providing a mechanistic link between sleep-disordered breathing and progressive episodic memory impairment. BBB breakdown and neuroinflammation are primary contributors to the pathway; MAM dysfunction is secondary to ischemic mitochondrial injury.

**Developmental Disorders.** Cerebral palsy and hypoxic–ischemic encephalopathy disrupt working memory through frontal–subcortical circuit injury [100], demonstrating that the convergent mechanisms operate across the lifespan from perinatal insult through late-life neurodegeneration. TGF-β1 pathway suppression during early developmental windows compounds dentate gyrus neurogenic failure, as discussed in Section 4.

**Brain Tumors.** Brain tumors impair memory through mass effect, edema, and seizure activity, while treatment amplifies the damage: radiation and chemotherapy add direct hippocampal injury, mitochondrial dysfunction, and chronic neuroinflammation to an already compromised circuit [101,102]. The therapeutic paradox, treating the tumor while injuring the hippocampus, makes neuroprotective strategies targeting convergent pathways a clinical priority, particularly for tumors near the temporal lobe, where hippocampal radiation exposure is unavoidable (Table 6).

## 7. Therapeutic Strategies

The mechanistic convergence of virus-associated hippocampal injury on four shared pathways has direct therapeutic implications. Pathogen-specific interventions, antiretrovirals, antivirals, and vaccines address the upstream trigger but leave the downstream molecular architecture largely intact. Cognitive recovery requires interventions that, in combination, restore MAM integrity, resolve chronic neuroinflammation, repair BBB function, and restore CREB-BDNF signaling, commensurate with the multi-pathway nature of the injury.

For HIV-associated neurocognitive disorder, antiretrovirals suppress viral replication but do not restore VAPB-PTPIP51 contact-site integrity, resolve microglial activation, or reverse the lipidomic signature of MAM disruption. A rational combination approach, which remains to be tested in controlled clinical trials, would pair antiretroviral therapy with a MAM stabilizing agent (sigma-1 receptor agonist or dasatinib-mediated Src inhibition to prevent PTPIP51 phosphorylation), a microglial modulator (masitinib, for which Phase II/III data exist in other neurological contexts), and aerobic exercise as the most accessible BDNF-enhancing intervention, targeting all four pathways simultaneously [4,57]. This combination strategy is mechanistically motivated but has not been evaluated in a controlled clinical trial in HAND; the evidence base for each component individually varies from preclinical only to Phase II clinical data.

For long COVID cognitive impairment, where no approved treatments exist, the inflammatory and vascular components of injury are most prominent. An anti-inflammatory backbone targeting complement dysregulation and microglial activation, combined with BBB repair strategies and aerobic exercise, addresses the dominant pathways. These approaches remain at the preclinical or early-phase clinical stage for long COVID specifically; the therapeutic rationale is derived from mechanistic overlap with better-studied neuroinflammatory conditions rather than from long COVID-specific trial data.

The role of aerobic exercise merits particular emphasis. Aerobic exercise consistently increases BDNF by 2–3-fold, enhances hippocampal neurogenesis, reduces neuroinflammatory markers, and improves BBB integrity through endothelial shear stress-mediated upregulation of tight junction proteins [7,8]. It engages all four convergent pathways simultaneously to a magnitude that no current pharmacological agent achieves as monotherapy, and it does so without the adverse-effect profile that limits dose escalation in most CNS-active drugs. In the absence of approved combination pharmacotherapy for post-viral hippocampal injury, aerobic exercise represents the most evidence-supported multi-pathway intervention currently available (Table 7).

## 8. Conclusions and Future Directions

Virus-associated memory impairment is not a collection of entirely distinct pathogen-specific syndromes. The evidence reviewed here is consistent with a convergent hippocampal failure framework driven by four shared molecular pathways, MAM dysfunction, chronic neuroinflammation, BBB disruption, and impaired CREB-BDNF signaling, through which multiple neurotropic and neurovirulent viruses engage through distinct upstream mechanisms. This framework is proposed as a unifying hypothesis that motivates combination therapeutic research rather than as an established biological fact; the strength of mechanistic evidence varies substantially across the nine pathogens discussed, and causal relationships between pathway activation and cognitive outcome have been rigorously established in some contexts (HIV, HSV-1) while remaining largely inferential in others (influenza, ZIKV, EBV).

This framework has several important implications. It provides a mechanistic explanation for why antiviral therapies, despite suppressing viral replication, leave substantial cognitive impairment unresolved; the downstream molecular injury operates independently of ongoing viral activity. It identifies genetic modulators, APOE4, BDNF Val66Met, TREM2, FKBP5, and sex-related biological factors that stratify individual vulnerability and can guide precision intervention design. It provides a biomarker framework, NfL, GFAP, p-tau217, albumin quotient, hippocampal subfield MRI, that enables pathway-matched rather than pathogen-matched mechanistic stratification, with the important caveat that cross-disorder validation for several of these tools remains incomplete.

Future research priorities include directly comparing MAM integrity across viral models using standardized assays, longitudinally characterizing biomarker trajectories in post-viral cognitive impairment to identify the window of maximal therapeutic opportunity, designing combination clinical trials that simultaneously target multiple convergent pathways with outcome measures sensitive to subfield-specific hippocampal function, and extending the framework to examine whether TGF-β1 pathway suppression represents a consistent co-contributor to dentate gyrus failure across viral contexts. The increasing burden of post-viral cognitive impairment, driven in particular by the long COVID epidemic, makes these research priorities urgent. The mechanistic framework developed here provides the scientific rationale for combination approaches that move beyond pathogen-specific treatment toward hippocampal circuit protection as a shared therapeutic goal, while acknowledging that translating this framework into clinical practice will require the rigorous multi-pathway trial evidence that does not yet exist (Table 8).

Memory is among the most complex biological phenomena in nature, spanning molecular events at individual synapses to systems-level circuit dynamics across timescales from milliseconds to decades and failing in ways that are simultaneously predictable in their anatomical pattern and deeply individual in their clinical expression. That conditions as etiologically distant as a trinucleotide repeat expansion, a retroviral infection, and a stress-related psychiatric disorder converge on the same hippocampal subfields through the same four molecular pathways is not a simplification of this complexity but a discovery within it.

## Figures and Tables

**Table 1:** Brain regions and their associated memory types, subtypes, temporal duration, and primary mnemonic functions. This table provides the neuroanatomical framework for understanding how diverse insults selectively damage specific memory systems. The hippocampal subfields (DG, CA3, CA1) bear the highest metabolic and synaptic demands and are the most consistently vulnerable across the 26 conditions reviewed. Legend: DG = Dentate Gyrus; CA1/CA3 = Cornu Ammonis subfields 1 and 3; dlPFC = Dorsolateral Prefrontal Cortex. Memory subtypes: Explicit (Declarative) = conscious, intentional recollection; Implicit = unconscious, automatic memory. Short-term = seconds to minutes; Long-term = hours to a lifetime; Ultra short-term = milliseconds to seconds; Relay/Consolidation = thalamic function supporting transfer between memory systems.

Brain Region	Memory Type	Memory Subtype	Short or Long-Term	Function
Hippocampus (DG)	Episodic, Spatial	Explicit	Long-term	Pattern separation; encoding distinct memories; reducing interference between similar experiences
Hippocampus (CA3)	Episodic, Associative	Explicit	Long-term	Associative retrieval; pattern completion from partial cues; recurrent collateral circuitry
Hippocampus (CA1)	Episodic, Temporal	Explicit	Long-term	Temporal binding; memory consolidation; integrating hippocampal and entorhinal signals
Entorhinal Cortex	Episodic, Spatial	Explicit	Long-term	Gateway to hippocampus; spatial mapping via grid cells; sensory input integration
Prefrontal Cortex (dlPFC)	Working, Executive	Explicit	Short-term	Holding and manipulating information online; strategic retrieval; decision-making
Amygdala	Emotional, Fear	Implicit/Explicit	Long-term	Emotional memory encoding; fear conditioning; modulation of hippocampal consolidation
Cerebellum	Procedural, Motor	Implicit	Long-term	Motor learning; timing and coordination; skill automation
Basal Ganglia/Striatum	Procedural, Habit	Implicit	Long-term	Habit formation; reward-based learning; cortico-striatal-thalamic circuit automation
Anterior Temporal Lobe	Semantic	Explicit	Long-term	Conceptual knowledge storage; word meaning; object and person recognition
Posterior Parietal Cortex	Spatial, Working	Explicit	Short-term	Spatial working memory; attention; visuospatial processing
Primary Sensory Cortices	Sensory	Implicit	Ultra short-term	Iconic (visual) and echoic (auditory) sensory buffer; perceptual continuity
Thalamus	Multiple	Explicit	Relay/Consolidation	Filtering and routing memory signals; thalamocortical relay; diencephalic memory consolidation
Mammillary Bodies	Episodic, Spatial	Explicit	Long-term	Spatial memory; memory consolidation via Papez circuit; thiamine-dependent function
Perirhinal Cortex	Semantic, Familiarity	Explicit	Long-term	Object recognition; familiarity-based memory judgments; semantic encoding

**Table 2:** Classification of memory types, subtypes, categories, and systems impaired across 17 neurodegenerative and cerebrovascular disorders. AD = Alzheimer’s Disease; PD = Parkinson’s Disease; LBD = Lewy Body Dementia; FTD = Frontotemporal Dementia; VaD = Vascular Dementia; PSP = Progressive Supranuclear Palsy; MSA = Multiple System Atrophy; ALS = Amyotrophic Lateral Sclerosis; NPH = Normal Pressure Hydrocephalus; HD = Huntington’s Disease; SCAs = Spinocerebellar Ataxias; CBS = Corticobasal Syndrome; CJD = Creutzfeldt–Jakob Disease; CTE = Chronic Traumatic Encephalopathy; PPA = Primary Progressive Aphasia; KS = Korsakoff Syndrome; FRDA = Friedreich’s Ataxia.

Disease	Type of Memory Impaired	Memory Subtype	Memory Category	Memory System
Alzheimer’s Disease (AD)	Episodic, Semantic	Explicit (Declarative)	Long-term Memory	Events, Facts
Parkinson’s Disease (PD)	Procedural, Episodic	Implicit, Explicit	Long-term Memory	Skills, Events
Lewy Body Dementia (LBD)	Episodic, Attention-based	Explicit, Implicit	Long-term Memory	Events, Facts, Tasks
Frontotemporal Dementia (FTD)	Semantic, Episodic	Explicit (Declarative)	Long-term Memory	Facts, Events
Vascular Dementia (VaD)	Episodic, Working	Explicit	Long-term, Short-term Memory	Events, Real-time Processing
Progressive Supranuclear Palsy (PSP)	Procedural, Episodic	Implicit, Explicit	Long-term Memory	Skills, Events
Multiple System Atrophy (MSA)	Procedural, Episodic	Implicit, Explicit	Long-term Memory	Skills, Events
Amyotrophic Lateral Sclerosis (ALS)	Working, Episodic	Explicit	Short-term, Long-term Memory	Events, Immediate Processing
Normal Pressure Hydrocephalus (NPH)	Episodic, Procedural	Explicit, Implicit	Long-term Memory	Events, Skills
Huntington’s Disease (HD)	Procedural, Episodic	Implicit, Explicit	Long-term Memory	Skills, Events
Spinocerebellar Ataxias (SCAs)	Spatial, Episodic	Explicit	Long-term Memory	Navigation, Events
Corticobasal Syndrome (CBS)	Procedural, Semantic	Implicit, Explicit	Long-term Memory	Skills, Facts
Creutzfeldt–Jakob Disease (CJD)	Episodic, Procedural	Explicit, Implicit	Long-term Memory	Events, Skills
Chronic Traumatic Encephalopathy (CTE)	Episodic, Working	Explicit	Long-term, Short-term Memory	Events, Immediate Processing
Primary Progressive Aphasia (PPA)	Semantic, Episodic	Explicit (Declarative)	Long-term Memory	Facts, Events
Friedreich’s Ataxia (FRDA)	Spatial, Episodic	Explicit (Declarative)	Long-term Memory	Navigation, Events
Korsakoff Syndrome (KS)	Anterograde, Retrograde	Explicit (Declarative)	Long-term Memory	Events, Facts

**Table 3:** Viral pathogens contribute to memory impairment through gene-environment interactions, direct neurotoxicity, chronic neuroinflammation, and vascular injury. Gene interactions indicate host genetic variants that modulate susceptibility or severity. ACE2, angiotensin-converting enzyme 2; APOE, apolipoprotein E; BBB, blood-brain barrier; BDNF, brain-derived neurotrophic factor; CMV, cytomegalovirus; DG, dentate gyrus; EBV, Epstein-Barr virus; EBNA1, EBV nuclear antigen 1; HSV-1, herpes simplex virus type 1; LTP, long-term potentiation; MBP, myelin basic protein; MS, multiple sclerosis; NLRP3, NOD-like receptor pyrin domain containing 3; SARS-CoV-2, severe acute respiratory syndrome coronavirus 2; TREM2, triggering receptor expressed on myeloid cells 2; VZV, varicella zoster virus; WNV, West Nile virus; ZIKV, Zika virus.

Virus	Genes Involved	Type of Memory Impaired	Primary Deficit	Primary Circuit/Region	Key Mechanisms
HSV-1	*apoE*, *app*, *bace1*, *psen1/2*, *mapt*	Episodic, Semantic	Encoding/Consolidation	Hippocampus, Entorhinal Cortex	Limbic tropism; promotes Aβ via ↑ β/γ-secretase; viral glycoproteins seed amyloid; APOE4 ↑ reactivation, impairs viral clearance
HIV	*apoE*, *trem2*, *creb*, *gsk3β*	Episodic, Working, Executive	Encoding/Consolidation	Hippocampus, Frontal–Striatal	Chronic neuroinflammation: Tat/gp120 inhibit CREB/BDNF; mitochondrial dysfunction; BBB disruption; premature aging
VZV	*apoE* (*risk modifier*)	Episodic, Semantic	Vascular/Inflammatory	Hippocampus, Vascular Networks	VZV vasculopathy; arteritis, thrombosis, ischemic/hemorrhagic stroke; white-matter lesions; prothrombotic state; post-herpetic inflammation
CMV	*apoE*, *trem2*, *bdnf* (*Val66Met*), *pi-calm*	Semantic, Working, Processing Speed	Executive/Retrieval	Frontal–Subcortical	Immunosenescence; CD8+ T-cell exhaustion/inflation; BDNF Met ↑ vulnerability; inflammaging; reduced T-cell diversity
EBV	*apoE*, *trem2*, *mapt*	Episodic, Working	Encoding/Autoimmune	Hippocampus, White Matter (MS)	Molecular mimicry (EBNA1/MBP, GFAP); autoimmune demyelination; anti-EBNA1 antibodies → cortical atrophy; 32× ↑ MS risk
Influenza	*apoE*, *ifnar*, *tlr3*, *nlrp33*, *mapk1*	Working, Attention, Semantic	Executive/Inflammatory	Hippocampus, Frontal Cortex	Microglial priming; cytokine storm (IL-6, TNF-α, IL-1β); impaired DG neurogenesis; exaggerated inflammatory responses to subsequent insults
SARS-CoV-2	*apoE*, *trem2*, *gsk3β*, *ace2*, *nlrp3*	Episodic, Working, Semantic, Attention	Encoding/Consolidation/ Executive	Hippocampus, Frontal, Parahippocampal, Olfactory	ACE2-mediated neuroinvasion (olfactory, BBB); microglial activation; NLRP3 inflammasome; vascular endothelial damage; complement-mediated synaptic pruning; “brain fog” in long COVID; APOE4 ↑ severity/cognitive sequelae
West Nile Virus (WNV)	*apoE*, *trem2*, *oas1* (*candidate*)	Episodic, Executive, Attention	Encoding/Consolidation	Hippocampus, Cortex, Thalamus, Brainstem	Direct neuronal infection (hippocampus, cortex); neuronal loss; persistent microglial activation; elevated IL-6/TNF-α; impaired LTP, DG neurogenesis, spatial memory
Zika Virus (ZIKV)	*apoE*, *axl*, *if-nar* (*candidate*)	Episodic, Working (emerging data)	Encoding/Neurogenesis Impairment	Hippocampus (DG neural progenitors)	Neurotropism for neural progenitor cells; impaired DG neurogenesis; microglial activation; congenital syndrome (microcephaly); adult cognitive sequelae emerging

Arrow facing up meaning increase (the expression of the protein increased).

**Table 4:** Classification of memory types, subtypes, categories, and systems impaired across acquired and developmental conditions. Gene(s) involved indicate genetic variants modulating susceptibility and severity. APOE, apolipoprotein E; BDNF, brain-derived neurotrophic factor; CR1, complement receptor 1; GSK3β, glycogen synthase kinase 3 beta; MAPT, microtubule-associated protein tau; TBI, traumatic brain injury; TREM2, triggering receptor expressed on myeloid cells 2.

Condition	Gene(s) Involved	Type of Memory Impaired	Memory Subtype	Memory Category	Memory System
Traumatic Brain Injury (TBI)	*apoE*, *mapt*, *trem2*, *gsk3β*, *bdnf*	Episodic, Working, Semantic	Explicit (Declarative)	Long-term Memory, Short-term Memory	Events, Facts, Working Memory
Sports Contact Injuries	*apoE*, *mapt*, *gsk3β*, *bdnf*, *cr1*	Episodic, Working	Explicit (Declarative)	Long-term Memory, Short-term Memory	Events, Facts, Working Memory
Accidents (Head Injuries)	*apoE*, *mapt*, *trem2*, *gsk3β*, *bdnf*	Episodic, Working, Semantic	Explicit (Declarative)	Long-term Memory, Short-term Memory	Events, Facts, Working Memory
Congenital Birth Deficits (e.g., Cerebral Palsy)	*GSK3β*, *apoE*, *mapt*, *bdnf*	Working, Semantic	Implicit, Explicit	Short-term Memory, Long-term Memory	Working Memory, Facts
Stroke	*apoE*, *mapt*, *trem2*, *gsk3β*, *bdnf*	Episodic, Semantic	Explicit (Declarative)	Long-term Memory	Events, Facts
Chronic Hypoxia (e.g., in preterm infants)	*apoE*, *mapt*, *gsk3β*, *bdnf*	Episodic, Working	Explicit (Declarative)	Long-term Memory, Short-term Memory	Events, Facts, Working Memory

**Table 5:** Key molecular players driving convergent hippocampal circuit failure across memory disorders. The primary function, conditions with the highest involvement, memory mechanisms, and therapeutic targeting strategies are shown for each gene or protein. AMPK, AMP-activated protein kinase; APOE, apolipoprotein E; BDNF, brain-derived neurotrophic factor; CBS, corticobasal syndrome; CREB, cAMP response element-binding protein; CTE, chronic traumatic encephalopathy; DG, dentate gyrus; FTD, frontotemporal dementia; GSK3β, glycogen synthase kinase 3 beta; HDAC, histone deacetylase; LATE, limbic-predominant age-related TDP-43 encephalopathy; LBD, Lewy body dementia; LTP, long-term potentiation; MAPT, microtubule-associated protein tau; NAD+, nicotinamide adenine dinucleotide; NMN, nicotinamide mononucleotide; NR, nicotinamide riboside; PDE4, phosphodiesterase 4; SIRT1, sirtuin 1; TDP-43, TAR DNA-binding protein 43; TREM2, triggering receptor expressed on myeloid cells 2; VaD, vascular dementia.

Gene	Primary Function	Conditions with Highest Involvement	Memory Mechanism	Therapeutic Targeting
*apoE*	Lipid transport, Aβ clearance, synaptic integrity	AD, LBD, VaD, HSV-1, HIV, CMV, TBI, Stroke	Synaptic repair, cholesterol delivery, blood–brain barrier integrity; APOE4 impairs Aβ clearance and enhances tau pathology	Anti-APOE4 antibodies; APOE mimetics; APOE gene editing; lipid-lowering strategies
*mapt* *(Tau)*	Microtubule stabilization, axonal transport	AD, FTD, PSP, CBS, CTE, TBI, HSV-1	Physiological tau regulates synaptic function; hyperphosphorylated tau disrupts transport, forms tangles, spreads trans-synaptically	Tau antisense oligonucleotides (BIIB080); anti-tau antibodies (semorinemab); GSK-3β/CDK5 inhibitors; MTBR tau vaccines
*creb*	Transcriptional regulation of memory genes	AD, PD, HD, HIV, Influenza (all conditions impairing LTP)	Master regulator of synaptic plasticity; phosphorylates to activate BDNF, c-fos, Arc; CREB dysfunction = impaired consolidation	PDE4 inhibitors (rolipram); HDAC inhibitors; AMPK activators (metformin); direct CREB activators
*bdnf*	Neurotrophin; synaptic plasticity, neurogenesis	AD, PD, HD, TBI, Stroke, CMV, Hypoxia	Activity-dependent secretion; TrkB signaling enhances LTP, spine maturation, DG neurogenesis; Val66Met polymorphism modulates secretion	Exercise (most effective); BDNF mimetics (7,8-dihydroxyflavone); TrkB agonists; ketamine (rapid BDNF induction)
*gsk3β*	Serine/threonine kinase regulating tau, CREB, inflammation	AD, FTD, PD, PSP, TBI, HIV, HCV	Hyperactive GSK3β phosphorylates tau (promotes tangles), inhibits CREB (blocks consolidation), activates NF-κB (inflammation) [84]	Lithium; tideglusib; AZD1080; small-molecule inhibitors (selective for GSK3β to avoid Wnt effects)
*trem2*	Microglial phagocytosis, inflammation regulation	AD, LBD, FTD, HSV-1, HIV, TBI	TREM2 variants impair microglial clearance of Aβ, tau, apoptotic debris; R47H variant increases AD risk 2–4-fold; loss of function causes Nasu- Hakola disease	TREM2 agonist antibodies (AL002); microglial activation modulators; anti-inflammatory strategies targeting DAP12 signaling
*sirt1*	NAD+-dependent deacetylase; longevity	AD, PD, HD, Aging	Deacetylates CREB (enhances transcription), p53 (reduces apoptosis), histones (chromatin remodeling); declines with age	NAD+ precursors (NMN, NR); resveratrol; SIRT1 activators (SRT1720); caloric restriction mimetics [85]
*p53*	Tumor suppressor; stress response	TBI, Stroke, Aging, Hypoxia	Regulates apoptosis, synaptic function; age-related DNA damage increases p53, shifting neurons from plasticity to survival	p53 inhibitors (pifithrin-α) in acute injury; SIRT1 activation to deacetylate p53; restoring SIRT1/p53 balance
*tdp-* *43*	RNA processing, local translation	ALS, FTD, LATE, Aging	Regulates synaptic mRNA transport/translation; mislocalization causes loss-of-function (impaired splicing) and gain-of-function (aggregates)	Antisense oligonucleotides; small molecules restoring nuclear localization; inhibitors of liquid–liquid phase separation

**Table 6:** Current FDA-approved therapies, active clinical trials (Phase 2/3, 2022 onwards), and investigational agents across major memory disorders. Mechanism of action and memory/cognitive impact are shown for approved therapies. Trial target and mechanism are shown for investigational agents. AChE, acetylcholinesterase; AD, Alzheimer’s disease; ALS, amyotrophic lateral sclerosis; ASO, antisense oligonucleotide; BBB, blood-brain barrier; CBS, corticobasal syndrome; CDR-SB, Clinical Dementia Rating Sum of Boxes; CJD, Creutzfeldt-Jakob disease; CSF, cerebrospinal fluid; CTE, chronic traumatic encephalopathy; DA, dopamine; DLB, dementia with Lewy bodies; FRDA, Friedreich’s ataxia; FTD, frontotemporal dementia; GLP-1, glucagon-like peptide-1; HD, Huntington’s disease; HTT, huntingtin; KS, Korsakoff syndrome; LP, lumboperitoneal; MAO-B, monoamine oxidase B; MAPK, mitogen-activated protein kinase; mAb, monoclonal antibody; MSA, multiple system atrophy; NMDA, N-methyl-D-aspartate; NPH, normal pressure hydrocephalus; Nrf2, nuclear factor erythroid 2-related factor 2; PD, Parkinson’s disease; PDD, Parkinson’s disease dementia; PDE, phosphodiesterase; PPA, primary progressive aphasia; PSP, progressive supranuclear palsy; RAGE, receptor for advanced glycation end products; SCAs, spinocerebellar ataxias; SOD1, superoxide dismutase 1; TREM2, triggering receptor expressed on myeloid cells 2; VA, ventriculoatrial; VaD, vascular dementia; VMAT2, vesicular monoamine transporter 2; VP, ventriculoperitoneal.

Disease	FDA-Approved Therapies	Mechanism	Memory/Cognitive Impact	Active Trials (Phase 2/3, 2022+)	Trial Target/Mechanism
AD	Donepezil, rivastigmine, galantamine	AChE inhibitor	Modest improvement in attention, working memory, episodic retrieval	Masitinib	Mast cell/neuroinflammation
	Memantine	NMDA antagonist	Stabilizes excitotoxicity; modest functional benefit	Lecanemab	Anti-Aβ mAb (approved 2023)
	Lecanemab [103]	Anti-Aβ mAb	Slows decline modestly (~27% CDR- SB); reduces plaques	Donanemab	Anti-Aβ mAb (approved 2024)
	Donanemab [104]	Anti-Aβ mAb	Slows decline ~35% (intermediate tau); reduces plaques/tau	BIIB080, IONIS-MAPTRx	Tau antisense oligonucleotide
				Semorinemab, tilavonemab	Anti-tau mAb
				GV-971 (sodium oligomannate)	Gut microbiome modulation
				Azeliragon (TTP488)	RAGE inhibitor, anti-inflammatory
PD/PDD	Levodopa, DA agonists	Dopamine replacement	Variable cognitive effect (dose-dependent “overdose”); improves motor	Prasinezumab, cinpanemab	Anti-α-synuclein mAb
	Rivastigmine (PDD approved)	AChE inhibitor	Improves attention, episodic retrieval in PDD	Nilotinib	Autophagy (c-Abl inhibitor)
				Exenatide	GLP-1 agonist, neuroprotection
DLB	Rivastigmine, donepezil	AChE inhibitor	Reduces cognitive fluctuations, improves attention	E2027	PDE9 inhibitor (cGMP pathway)
				Neflamapimod	p38α MAPK inhibitor, anti-inflammatory
FTD	None approved	—	—	ABBV-8E12, tilavonemab	Anti-tau mAb
				TPN-101 (troriluzole)	Glutamate modulator, neuroprotection
				AL001	Anti-sortilin (progranulin pathway)
HD	Tetrabenazine, deutetrabenazine	VMAT2 inhibitor (motor only)	No cognitive benefit; may worsen depression	Branaplam (LMI070)	Splicing modulator (↑ HTT)
				Pridopidine	Sigma-1 receptor agonist, neuroprotection
				WVE-003	HTT-lowering ASO (allele-selective)
VaD	No specific approval	—	Stroke prevention (antiplatelet, statin, BP control) limits progression	Cilostazol	PDE3 inhibitor, antiplatelet, vasodilator
	Donepezil (off label)	AChE inhibitor	Modest benefit in mixed dementia	NA-1 (Tat-NR2B9c)	NMDA antagonist, neuroprotection (stroke)
PSP	None approved	—	—	Tilavonemab, ABBV-8E12	Anti-tau mAb
				AL001	Anti-sortilin (tau clearance)
				Davunetide (failed Ph3)	Microtubule stabilizer
MSA	None approved	—	—	Rasagiline + riluzole	MAO-B inhibitor + glutamate modulator
				Rifampicin	α-Synuclein aggregation inhibitor
ALS	Riluzole	Glutamate modulator	No cognitive benefit; slows motor decline modestly	AMX0035 (sodium phenylbutyrate-taurursodiol)	Mitochondrial/ER stress
	Edaravone	Antioxidant	No cognitive benefit; slows motor decline modestly	Tofersen (approved 2023, SOD1)	SOD1 ASO
	Tofersen, (SOD1-ALS) [105]	SOD1 ASO	Slows motor decline in SOD1 mutation carriers	Verdiperstat	Myeloperoxidase inhibitor, anti-inflammatory
NPH	CSF shunting (VP, VA, LP shunt)	CSF diversion	Improves gait, cognition (attention, executive) in 60–80%	—	—
SCAs	None approved	—	—	Troriluzole	Glutamate modulator
				AAV-frataxin gene therapy (FRDA)	Gene replacement (FRDA only)
CBS	None approved	—	—	ABBV-8E12, tilavonemab	Anti-tau mAb
CJD	Noneffective	—	—	Quinacrine, pentosan polysulfate	Prion aggregation inhibitors (compassionate use, not effective)
CTE	None approved	—	Symptomatic management only	—	—
PPA	None approved	—	Speech therapy mainstay	GV-971, anti-tau (lvPPA/AD pathology)	See AD trials (lvPPA overlaps with AD)
FRDA	None approved	—	—	Omaveloxolone (approved 2023)	Nrf2 activator, mitochondrial antioxidant
				AAV-frataxin gene therapy	Gene replacement
KS	Thiamine replacement (preventive/acute)	Restores thiamine-dependent metabolism	Prevents progression if given early; limited reversal once chronic	—	—

Arrow pointing up means increase.

**Table 7:** Disease-specific memory deficits, primary cognitive failure modes, neuroanatomical circuits, and genetic drivers across neurodegenerative and cerebrovascular disorders. AD, Alzheimer’s disease; ALS, amyotrophic lateral sclerosis; APOE, apolipoprotein E; APP, amyloid precursor protein; ATXN, ataxin; C9orf72, chromosome 9 open reading frame 72; CACNA1A, calcium voltage-gated channel subunit alpha 1A; CBS, corticobasal syndrome; CBD, corticobasal degeneration; CJD, Creutzfeldt-Jakob disease; COQ2, coenzyme Q2; CTE, chronic traumatic encephalopathy; DM, diabetes mellitus; FTD, frontotemporal dementia; FTLD, frontotemporal lobar degeneration; FUS, fused in sarcoma; FXN, frataxin; GBA, glucocerebrosidase; GRN, granulin; HD, Huntington’s disease; HTN, hypertension; HTT, huntingtin; KS, Korsakoff syndrome; LBD, Lewy body dementia; LRRK2, leucine-rich repeat kinase 2; MAPT, microtubule-associated protein tau; MSA, multiple system atrophy; NPH, normal pressure hydrocephalus; PARK2, parkin; PINK1, PTEN-induced kinase 1; PD, Parkinson’s disease; PPA, primary progressive aphasia; PRNP, prion protein; PSEN1/2, presenilin 1/2; PSP, progressive supranuclear palsy; SCAs, spinocerebellar ataxias; SNCA, alpha-synuclein; SOD1, superoxide dismutase 1; TARDBP, TAR DNA-binding protein; TREM2, triggering receptor expressed on myeloid cells 2; TDP, TAR DNA-binding protein; VaD, vascular dementia; bv, behavioral variant; lv, logopenic variant; nfv, nonfluent variant; SD, semantic dementia; sv, semantic variant.

Disease	Type of Memory Impaired	Primary Deficit	Primary Circuit/Region	Main Gene(s)
AD	Episodic → Semantic, Working	Encoding/Consolidation	Hippocampus/Entorhinal → Neocortex	APP, PSEN1/2, APOE ε4, TREM2
PD	Working, Procedural, Episodic (retrieval)	Retrieval/ Executive	Striatum, Frontal–Striatal Loops	SNCA, LRRK2, PARK2, PINK1, GBA
LBD	Episodic, Working, Attention	Encoding/ Attention	Hippocampus, Posterior Cortex, Frontal	SNCA, GBA, APOE
FTD-bv	Working, Executive, Episodic (encoding)	Strategic Encoding	Frontal–Subcortical Networks	MAPT, GRN, C9orf72
FTD-SD	Semantic (severe), Episodic (variable)	Semantic Knowledge	Anterior Temporal Lobes	MAPT, GRN, C9orf72
VaD	Working, Executive, Episodic (retrieval)	Retrieval/ Processing Speed	Frontal–Subcortical, White Matter	Vascular risk factors (HTN, DM)
PSP	Working, Executive, Procedural	Retrieval/Executive	Basal Ganglia, Frontal, Brainstem	MAPT (H1 haplotype)
MSA	Executive, Working (mild)	Executive/Procedural	Striatum, Cerebellum, Brainstem	COQ2 (rare), SNCA (pathology)
ALS	Working, Executive (ALS-FTD)	Executive/Working	Frontal Cortex (in ALS-FTD)	SOD1, TARDBP, C9orf72, FUS
NPH	Episodic (retrieval), Procedural	Retrieval/Psychomotor	Frontal–Subcortical (compression)	Not genetic
HD	Procedural, Working, Episodic (retrieval)	Retrieval/Executive	Striatum, Frontal–Striatal Loops	HTT (CAG expansion)
SCAs	Working, Procedural, Episodic (retrieval)	Retrieval/Executive	Cerebellum, Cerebellar–Frontal Circuits	ATXN1/2/3, CACNA1A (SCA6)
CBS	Working, Visuospatial, Episodic (retrieval)	Retrieval/Executive	Asymmetric Frontoparietal	Variable (CBD, PSP, AD, FTLD)
CJD	All systems (rapid global)	Global Network Failure	Widespread (Cortex, Thalamus, Cerebellum)	PRNP (genetic), prion propagation
CTE	Episodic, Working, Executive	Encoding/Executive	Perivascular Frontal/Temporal	APOE (risk modifier), tau pathology
PPA-nfv	Verbal (language-dependent)	Phonological Encoding	Left Inferior Frontal/Insula	GRN, MAPT (FTLD-tau/TDP)
PPA-sv	Semantic (severe), Verbal	Semantic Knowledge	Anterior Temporal Lobes	GRN, MAPT
PPA-lv	Episodic, Verbal Working	Consolidation (AD pathology)	Left Temporoparietal, Hippocampus	APOE ε4 (AD pathology)
FRDA	Working, Procedural, Episodic (retrieval)	Retrieval/Executive	Cerebellum, Cerebellar–Frontal Circuits	FXN (GAA expansion)
KS	Anterograde, Retrograde	Consolidation	Diencephalic-Hippocampal (Mammillary Bodies, Thalamus)	Thiamine deficiency

**Table 8:** Convergent molecular mechanisms—neuroinflammation, mitochondria-associated membrane dysfunction, blood–brain barrier disruption, and impaired CREB-BDNF signaling—across 26 neurological, viral, psychiatric, and acquired conditions. ✓ = documented involvement; — = not established or minimal evidence. Memory type impaired and the primary brain region affected are shown for each condition. BBB, blood–brain barrier; BDNF, brain-derived neurotrophic factor; CA1/CA3, cornu ammonis subfields; CREB, cAMP response element-binding protein; DG, dentate gyrus; MAM, mitochondria-associated membrane; ALS, amyotrophic lateral sclerosis; CBS, corticobasal syndrome; CJD, Creutzfeldt–Jakob disease; CMV, cytomegalovirus; CTE, chronic traumatic encephalopathy; EBV, Epstein–Barr virus; FRDA, Friedreich’s ataxia; HAND, HIV-associated neurocognitive disorder; HSV-1, herpes simplex virus type 1; MSA, multiple system atrophy; MS, multiple sclerosis; NPH, normal pressure hydrocephalus; PPA, primary progressive aphasia; PSP, progressive supranuclear palsy; SARS-CoV-2, severe acute respiratory syndrome coronavirus 2; SCAs, spinocerebellar ataxias; VZV, varicella zoster virus; WNV, West Nile virus; ZIKV, Zika virus.

Disease/Condition	BBB	MAM	Neuro-inflammation	CREB-BDNF	Memory Type Impaired	Brain Region
Alzheimer’s Disease	✓	✓	✓	✓	Episodic, Semantic, Working	Hippocampus (DG, CA1), Entorhinal Cortex
Parkinson’s Disease	✓	✓	✓	✓	Working, Episodic (late)	Substantia Nigra, Frontostriatal, CA1 (late)
Lewy Body Dementia	✓	✓	✓	✓	Episodic (retrieval), Working	CA1, Brainstem, Limbic Cortex
Frontotemporal Dementia	✓	✓	✓	✓	Semantic, Episodic (retrieval)	Frontal, Anterior Temporal, DG, CA1
Huntington’s Disease	—	✓	✓	✓	Working, Episodic (late)	Striatum, Frontostriatal, Hippocampus (late)
Vascular Dementia	✓	—	✓	✓	Episodic, Working	CA1, White Matter, Frontal
PSP	✓	✓	✓	—	Working, Procedural	Frontostriatal, Brainstem, Thalamus
MSA	✓	✓	✓	—	Working, Procedural	Cerebellum, Striatum, Brainstem
ALS	✓	✓	✓	✓	Working, Episodic	Frontal, Hippocampus
NPH	✓	—	✓	—	Working, Episodic	Frontostriatal, Hippocampus
SCAs	—	✓	✓	✓	Working, Procedural	Cerebellum, Frontal
CBS	✓	✓	✓	—	Working, Episodic	Frontal, Parietal, Hippocampus
CJD	✓	✓	✓	—	Episodic, Semantic, Working	Widespread, Hippocampus, Cortex
CTE	✓	✓	✓	✓	Episodic, Working	Hippocampus, Frontal, CA1
PPA	✓	—	✓	✓	Semantic, Working	Anterior Temporal, Frontal
FRDA	—	✓	✓	✓	Working, Procedural	Cerebellum, Spinal Cord, Frontal
Korsakoff Syndrome	✓	✓	✓	✓	Episodic, Working	Hippocampus, Thalamus, Mammillary Bodies
HSV-1	✓	—	✓	✓	Episodic (anterograde/retrograde)	Hippocampus, Entorhinal Cortex, Limbic
HIV/HAND	✓	✓	✓	✓	Episodic, Working, Processing Speed	DG, CA1, CA3, Frontal
VZV	✓	—	✓	—	Episodic, Executive	Hippocampus, Cerebral Arteries, White Matter
CMV	—	—	✓	✓	Episodic, Working	Hippocampus, Frontal
EBV/MS	✓	—	✓	✓	Episodic, Working, Processing Speed	Hippocampus, White Matter, Frontotemporal
Influenza	✓	—	✓	✓	Episodic, Attention, Executive	Hippocampus, Frontal
SARS-CoV-2/Long COVID	✓	✓	✓	✓	Episodic, Attention, Executive	Hippocampus, Parahippocampus, DG, CA1
WNV	✓	—	✓	—	Episodic, Executive	Hippocampus, Frontal
ZIKV	—	—	✓	✓	Spatial, Episodic	DG, Hippocampus

## Data Availability

No new data were created or analyzed in this study.

## Abbreviations

Aβ	Amyloid beta
AD	Alzheimer’s disease
ALS	Amyotrophic lateral sclerosis
APOE4	Apolipoprotein E ε4 allele
ATP	Adenosine triphosphate
BBB	Blood–brain barrier
BDNF	Brain-derived neurotrophic factor
CA1/CA3	Cornu Ammonis hippocampal subfields
CBS	Corticobasal syndrome
CDR-SB	Clinical Dementia Rating–Sum of Boxes
CJD	Creutzfeldt–Jakob disease
CMV	Cytomegalovirus
CNS	Central nervous system
CREB	cAMP response element-binding protein
CTE	Chronic traumatic encephalopathy
DaTscan	Dopamine transporter imaging
DG	Dentate gyrus
EBV	Epstein–Barr virus
EEG	Electroencephalography
ER	Endoplasmic reticulum
FRDA	Friedreich’s ataxia
FTD	Frontotemporal dementia
GFAP	Glial fibrillary acidic protein
HAND	HIV-associated neurocognitive disorder
HD	Huntington’s disease
HIV	Human immunodeficiency virus
HSV-1	Herpes simplex virus type 1
IL	Interleukin
KS	Korsakoff syndrome
LBD	Lewy body dementia
LTP	Long-term potentiation
MAM	Mitochondria-associated membrane
MCI	Mild cognitive impairment
MRI	Magnetic resonance imaging
MSA	Multiple system atrophy
NAC	N-acetylcysteine
NLRP3	NOD-like receptor pyrin domain-containing 3
NPH	Normal pressure hydrocephalus
PD	Parkinson’s disease
PET	Positron emission tomography
PPA	Primary progressive aphasia
PSP	Progressive supranuclear palsy
PTSD	Post-traumatic stress disorder
SCAs	Spinocerebellar ataxias
SARS-CoV-2	Severe acute respiratory syndrome coronavirus 2
TBI	Traumatic brain injury
TNF-α	Tumor necrosis factor alpha
VaD	Vascular dementia
VAPB	Vesicle-associated membrane protein-associated protein B
VZV	Varicella zoster virus
WNV	West Nile virus
ZIKV	Zika virus
